# The Feasibility of Using CT-Guided ROI for Semiquantifying Striatal Dopamine Transporter Availability in a Hybrid SPECT/CT System

**DOI:** 10.1155/2014/879497

**Published:** 2014-11-02

**Authors:** Chien-Chin Hsu, Yen-Hsiang Chang, Wei-Che Lin, Shu-Wen Tang, Pei-Wen Wang, Yung-Cheng Huang, Nan-Tsing Chiu

**Affiliations:** ^1^Department of Nuclear Medicine, Kaohsiung Chang Gung Memorial Hospital, Chang Gung University College of Medicine, 123 Dapi Road, Niaosong District, Kaohsiung City 83301, Taiwan; ^2^Department of Diagnostic Radiology, Kaohsiung Chang Gung Memorial Hospital, Chang Gung University College of Medicine, 123 Dapi Road, Niaosong District, Kaohsiung City 83301, Taiwan; ^3^Department of Nuclear Medicine, National Cheng Kung University Hospital, College of Medicine, National Cheng Kung University, 138 Shengli Road, Tainan City 70428, Taiwan

## Abstract

A hybrid SPECT/CT system provides accurate coregistration of functional and morphological images. CT-guided region of interest (ROI) for semiquantifying striatal dopamine transporter (DAT) availability may be a feasible method. We therefore assessed the intra- and interobserver reproducibility of manual SPECT and CT-guided ROI methods and compared their semiquantitative data with data from MRI-guided ROIs. We enrolled twenty-eight patients who underwent Tc-99m TRODAT-1 brain SPECT/CT and brain MRI. ROIs of the striatal, caudate, putamen, and occipital cortex were manually delineated on the SPECT, CT, and MRI. ROIs from CT and MRI were transferred to the coregistered SPECT for semiquantification. The striatal, caudate, and putamen nondisplaceable binding potential (BP_ND_) were calculated. Using CT-guided ROIs had higher intra- and interobserver concordance correlation coefficients, closer Bland-Altman biases to zero, and narrower limits of agreement than using manual SPECT ROIs. The correlation coefficients of striatal, caudate, and putamen BP_ND_ were good between manual SPECT and MRI-guided ROI methods and even better between CT-guided and MRI-guided ROI methods. Conclusively, CT-guided ROI delineation for semiquantifying striatal DAT availability in a hybrid SPECT/CT system is highly reproducible, and the semiquantitative data correlate well with data from MRI-guided ROIs.

## 1. Introduction

Dopamine transporter (DAT) imaging can detect presynaptic dopamine neuronal dysfunction and is useful in the differentiation between conditions with and without presynaptic dopaminergic deficit [1–3]. Currently, single photon emission computed tomography (SPECT) radioligands for DAT, such as I-123 *β*-CIT (2*β*-carboxymethoxy-3*β*-(4-iodophenyl)tropane), I-123 FP-CIT (N-*ω*-fluoropropyl-2*β*-carbomethoxy-3*β*-(4-iodophenyl)nortropane), and Tc-99m TRODAT-1 ([2-[[2-[[[3-(4-chlorophenyl)-8-methyl-8-azabicyclo[3,2, 1]oct-2-yl]methyl](2-mercaptoethyl)-amino]ethyl]amino]ethanethiolato(3-)-*N*2,*N*2′,*S*2,*S*2′]oxo-[1*R*-(exo-exo)]), have been available for daily clinical practice [4, 5]. For interpretation of DAT-SPECT, using visual assessment together with semiquantitative data from region of interest (ROI) or volume of interest (VOI) has been recommended by Nuclear Medicine Associations [6, 7]. Semiquantification can provide more objective information and reinforce the visual assessment [8]. If done rigorously, the semiquantitative data may increase diagnostic accuracy, provide early disease detection, and also allow disease progression to be assessed [9].

Several approaches for semiquantifying striatal DAT availability have been developed [10]. Semiautomated and fully automated software packages save time and have better reproducibility [11–14]. However, they may not be available. Moreover, different programs vary in their image processing algorithms, and the automated VOI placement should be visually verified, especially in patients with advanced disease or abnormal anatomy [7]. Human-observer-dependent ROI technique is still the recommended method for daily clinical practice [8]. The conventional manual SPECT ROI method is simple but often associated with considerable interobserver variability. Using a standard ROI template can reduce variability, but individual striatal sizes, shapes, and positions are not always the same. Combining individual brain magnetic resonance imaging (MRI) and SPECT to facilitate ROI delineation is an acceptable method in research studies [15, 16]. However, brain MRI is not routinely acquired for patients undergoing DAT-SPECT in clinical practice. Furthermore, misregistration may occur while separately acquired DAT-SPECT and brain MRI are fused.

At present, hybrid technology with a SPECT/CT system allows sequential acquisition of SPECT and CT in a single study without requiring a change in patient position [17, 18]. It enables more accurate coregistration of functional and morphological images. For semiquantifying striatal DAT availability, delineating ROIs based on the coregistered CT image might be a feasible method. There has been a study using Tc-99m TRODAT-1 SPECT/CT to evaluate patients with Tourette's syndrome [19]. They drew ROIs on the striatum and cerebellum under the help of anatomic integration of CT images for semiquantitative analysis. With the increasing availability of hybrid SPECT/CT devices, CT-guided ROI delineation for semiquantifying striatal DAT availability has potential to become a recommended method in daily clinical practice. To evaluate the feasibility, we retrospectively assessed the intra- and interobserver reproducibility of semiquantitative data from manual SPECT and CT-guided ROI methods. We also compared the semiquantitative data with data from MRI-guided ROIs.

## 2. Materials and Methods

### 2.1. Patients

Between January and December 2011, 28 patients (15 men, 13 women; age range, 46–79 years; mean age ± standard deviation (SD), 67.7 ± 8.7 years) who underwent Tc-99m TRODAT-1 brain SPECT/CT and brain MRI within six months were enrolled in this retrospective study. The study was approved by our hospital's Institutional Review Board with a waiver of consent.

### 2.2. Tc-99m TRODAT-1 Brain SPECT/CT

Each patient was intravenously injected with a 925-MBq (25 mCi) dose of Tc-99m TRODAT-1 (Institute of Nuclear Energy Research, Lung-Tan, Taiwan). The brain SPECT and CT scans were done 4 h later consecutively, using a hybrid SPECT/CT system (Symbia T; Siemens Medical Solutions, Hoffman Estate, Illinois, USA) with patients lying stably in a supine position with a head holder. The SPECT/CT system integrates a dual-head SPECT camera with a two-slice spiral CT installed within the same gantry. SPECT images were obtained with 30 s per step acquiring 120 projections over a circular 360° rotation using low-energy, high-resolution parallel-hole collimators. A 128 × 128 matrix and a ×1.45 zoom were used. The CT images were acquired without contrast medium; they used the following parameters: 130 kV; 45 mAs (Image Quality Reference mAs, CARE Dose 4D; Siemens Medical Solutions); rotation time, 1.5 s; collimation, 2 × 2.5 mm.

### 2.3. MRI of Brain

All brain MRI were done on a 3.0T MRI scanner (Signa; GE Medical Systems, Milwaukee, WI, USA) with an eight-channel head coil. Each patient's head was immobilized with cushions inside the head coil in order to avoid movement problems. T2-weighted transaxial images were used for coregistration with SPECT images. The T2-weighted images were obtained using the following parameters: TR = 4200 ms; TE = 102 ms; FOV = 240 × 240 mm; matrix size = 320 × 256; number of slices = 20; and 5-mm thick slices.

### 2.4. Images Reconstruction, Fusion, and ROI Delineation

CT images were reconstructed to a 512 × 512 image matrix with a very smooth kernel, H08s (Siemens Medical Solutions), for SPECT attenuation correction, and a medium smooth kernel, H30s (Siemens Medical Solutions), for image fusion and ROI delineation. Raw SPECT data were reconstructed into transaxial slices using flash 3D (OSEM reconstruction method with 3D collimator beam modeling) with 8 subsets and 8 iterations and corrected with the H08s CT attenuation map. Images were smoothed using a 3D spatial Gaussian filter (full width at half maximum, 6 mm). The reconstructed transaxial slice thickness was 3.3 mm. Reorientation with sagittal slices parallel to the anterior commissure-posterior commissure (AC-PC) line and correction for transaxial and coronal slices deviation was done manually by inspecting the SPECT images. Three consecutive SPECT transaxial slices showing the highest striatal uptake were summed. For manual SPECT ROI delineation, ROIs of the striatum, caudate, putamen, and occipital cortex were manually delineated directly on the summed SPECT image.

**Figure 1:**
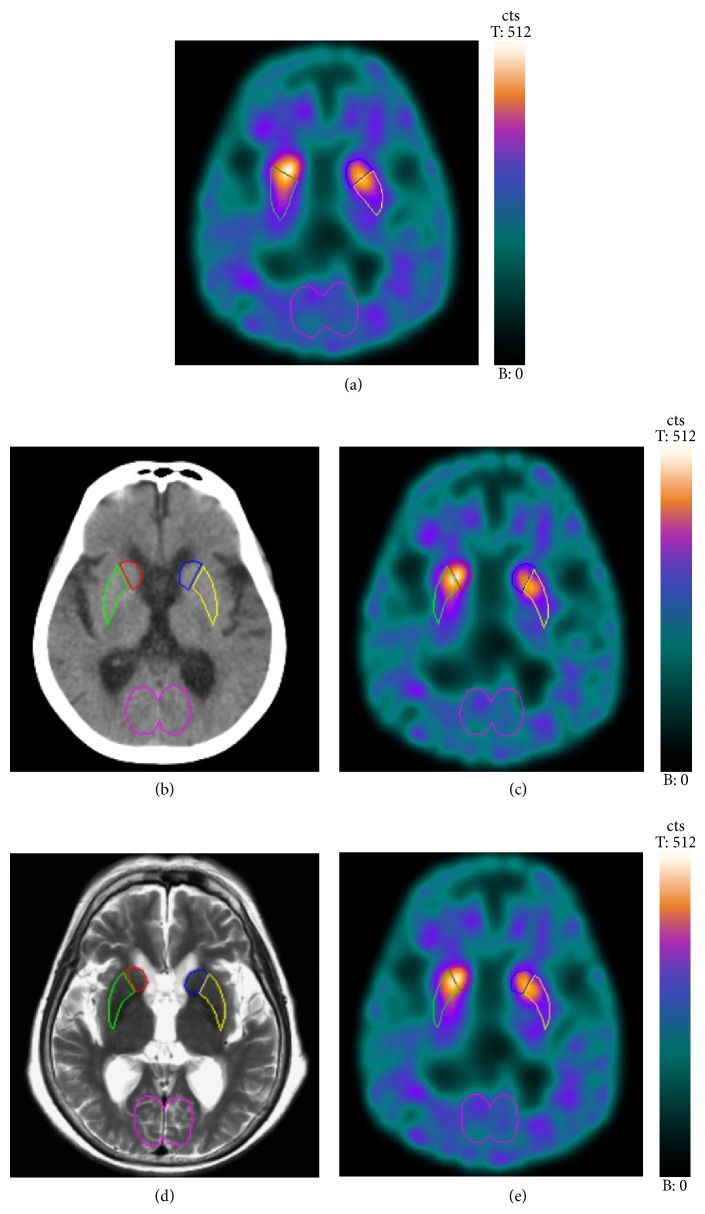
Regions of interest (ROIs) were manually drawn directly on the summed SPECT image (a). In CT-guided method, ROIs were manually delineated directly on one CT slice with the best recognizable striatum. ROIs were then transferred to the hardware-based coregistered summed SPECT image (b, c). In MRI-guided method, ROIs were manually delineated directly on one MRI slice with the best recognizable striatum. ROIs were then transferred to the software-based coregistered summed SPECT image (d, e).

The SPECT and CT images coregistration was done using an autoregistration model on manufacturer's nuclear medicine workstation (Syngo MI workplace, version VA60B; Siemens Healthcare). Reorientation was also done manually by inspecting the fused SPECT/CT images with identical parameters (*x*, *y*, *z*) for SPECT and CT images. Three consecutive SPECT transaxial slices showing the highest striatal uptake were summed. One CT slice with the most recognizable striatum was chosen for ROI drawing. CT-guided ROIs of the striatum, caudate, putamen, and occipital cortex were manually delineated directly on the CT image (Figure 1(b)). The observers were blinded to SPECT or fused SPECT/CT images when drawing CT-guided ROIs. Some anatomical landmarks can be identified on the CT image for accurate ROI delineation. The head of caudate nucleus is easily located because it bulges into the frontal horn of the lateral ventricle. After the bilateral heads of the caudate nucleus have been identified, the relatively hypodense right and left internal capsule can be recognized by its special “>” and “<” shape. The caudate nucleus and thalamus are medial to the internal capsule, and the lentiform nucleus is lateral to the internal capsule. The lentiform nucleus is wedge-shaped. The narrow part of the wedge is occupied by the globus pallidus and the putamen forms the lateral portion of the lentiform nucleus. Because the external capsule and the lamina of nerve fibers that separate putamen from the globus pallidus cannot be well identified on CT images, the ROI delineation for putamen requires knowledge of their anatomical positions. These CT-guided ROIs were then transferred to the coregistered summed SPECT image (Figure 1(c)).

To assess the intra- and interobserver reproducibility, these processing steps of manual SPECT and CT-guided ROI delineation were done twice by one experienced nuclear medicine physician and once by another nuclear medicine physician.

For MRI-guided ROI delineation, SPECT images were first coregistered automatically with T2-weighted MRI images using a 3D volume fusion which employs an algorithm based on normalized mutual information on the same workstation (Syngo MI workplace, version VA60B; Siemens Healthcare). Then the observer visually verified and adjusted manually the fused SPECT/MRI in axial, coronal, and sagittal planes for the best image registration. Reorientation was also done manually by inspecting the fused SPECT/MRI with identical parameters (*x*, *y*, *z*) for SPECT and MRI. Three consecutive SPECT transaxial slices showing the highest striatal uptake were summed. One MRI slice with the most recognizable striatum was chosen for ROI drawing. MRI-guided ROIs of the striatum, caudate, putamen, and occipital cortex were manually delineated directly on the MRI image (Figure 1(d)). The observers were blinded to SPECT or fused SPECT/MRI images when drawing MRI-guided ROIs. These MRI-guided ROIs were then transferred to the coregistered summed SPECT image (Figure 1(e)).

### 2.5. Semiquantifying Striatal and Subregional TRODAT-1 BP_ND_

We used the low DAT concentration area of occipital cortex as a background ROI. The striatal, caudate, and putamen nondisplaceable binding potential (BP_ND_) were calculated by subtracting the mean counts of the occipital cortex ROI from the mean counts of their ROIs and dividing the result by the mean counts of the occipital cortex ROI [20]. Because not all patients' final diagnoses were available, we used visual rating as standard for evaluating the accuracy of BP_ND_ measured by manual SPECT and CT-guided ROI methods. Two experienced nuclear medicine physicians who were blinded to clinical or semiquantitative data visually assessed the SPECT images independently to render their diagnosis. The caudate and putamen uptake were rated dichotomously as “normal” and “pathological.” Disagreements were resolved in discussion to reach a consensus.

### 2.6. Measurement of Radiation Dose in CT

For each patient, the volume-weighted CT dose index (CTDIvol) and dose length product (DLP) were available in the patient protocol and were recorded. DLP is the product of CTDIvol (mGy) and scan length (cm). The DLP (mGy cm) was then multiplied with a head-specific conversion factor, 0.0021 (mSv mGy^−1^ cm^−1^) [21]. This product yielded the effective dose (mSv) from CT.

### 2.7. Statistical Analysis

Continuous variables are expressed as mean ± SD. The intra- and interobserver reproducibility of manual SPECT and CT-guided ROI methods were assessed by Lin's concordance correlation coefficient and Bland-Altman (BA) analysis [22, 23]. The following descriptive scale was used for strength of agreement of concordance correlation coefficient: value > 0.99 as almost perfect, 0.95–0.99 as substantial, 0.90–0.95 as moderate, and < 0.90 as poor agreement [24]. The BA bias is the mean of difference and BA limits of agreement are the interval of bias ±1.96 SD. The semiquantitative data from manual SPECT and CT-guided ROIs were also compared with data from MRI-guided ROIs by Pearson's correlation and linear regression. Receiver operating characteristic (ROC) curve analysis was performed to evaluate and compare the diagnostic accuracy of the two methods. MedCalc 12.1 (MedCalc Software, Mariakerke, Belgium) was used for all statistical analyses. Significance was set at *P* < 0.05.

## 3. Results

The intra- and interobserver concordance correlation coefficients, BA biases, and limits of agreement of manual SPECT and CT-guided ROI methods were shown in Table 1. Using manual SPECT ROI method, the intraobserver agreement was substantial for striatal and caudate BP_ND_ and moderate for putamen BP_ND_, whereas the interobserver agreement was poor for striatal, caudate, and putamen BP_ND_. Using CT-guided ROI method, the intra- and interobserver agreement were substantial for striatal, caudate, and putamen BP_ND_. The BA analysis also showed that the semiquantitative data from CT-guided ROIs had closer intra- and interobserver biases to zero and narrower intra- and interobserver limits of agreement.

**Figure 2:**
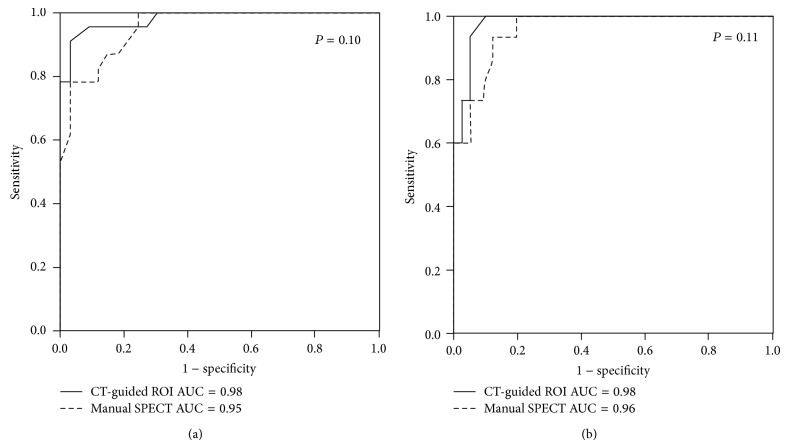
Receiver operating characteristic curve analysis for comparing the accuracy of caudate BP_ND_ (a) and putamen BP_ND_ (b) from manual SPECT and CT-guided ROI methods.

ROC curve analysis was performed to evaluate and compare the diagnostic accuracy of caudate and putamen BP_ND_ from the two methods (Figure 2). For caudate uptake, the areas under the curve (AUCs) of manual SPECT and CT-guided ROI methods were 0.95 ± 0.02 and 0.98 ± 0.01, respectively (*P* = 0.10). For putamen uptake, the AUCs of manual SPECT and CT-guided ROI methods were 0.96 ± 0.02 and 0.98 ± 0.01, respectively (*P* = 0.11).

**Figure 3:**
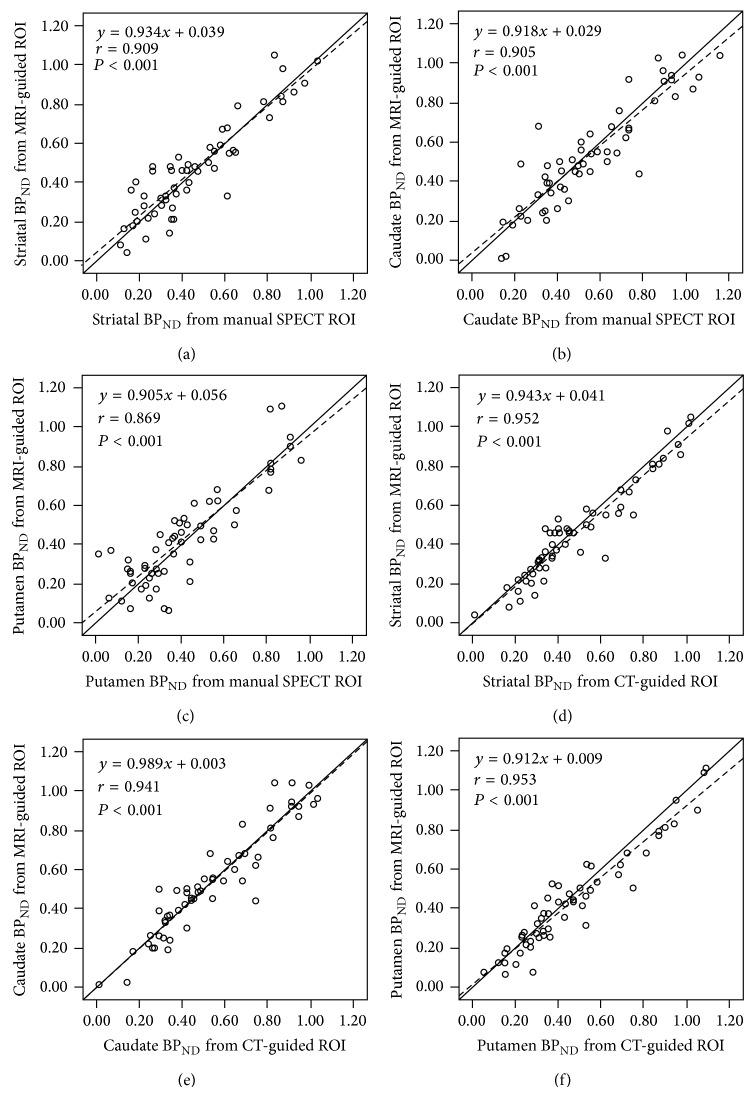
Scatter plots and regression lines (dashed line) of striatal, caudate, and putamen nondisplaceable binding potential (BP_ND_) between manual SPECT and MRI-guided regions of interest (ROIs) (a–c) and between CT-guided and MRI-guided ROIs (d–f).

Scatter plots and regression lines of striatal, caudate, and putamen BP_ND_ between the two methods and MRI-guided ROIs were shown in Figure 3. The correlation coefficients of striatal, caudate, and putamen BP_ND_ were good (*r* = 0.91, 0.91, and 0.87, respectively, all *P* < 0.01, Figures 3(a)–3(c)) between manual SPECT and MRI-guided ROI methods and even better (*r* = 0.95, 0.94, and 0.95, respectively, all *P* < 0.01, Figures 3(d)–3(f)) between CT-guided and MRI-guided ROI methods. The better correlation of CT-guided ROI than manual SPECT ROI was found in patients with low putamen BPND (Figures 3(c), and 3(f)). In patients with putamen BP_ND_ less than 0.53, as this value is the mean value minus 2 SD of healthy control group in a previous study [16], the correlation coefficient between CT-guided and MRI-guided ROI methods was 0.83 (*P* < 0.01) and that between manual SPECT and MRI-guided ROI methods was only 0.57 (*P* < 0.01).

The additional radiation exposure from CT ranged from 0.31 mSv to 0.50 mSv (mean ± SD, 0.36 ± 0.04 mSv) for the 28 patients.

## 4. Discussion

For clinical practice, excellent reproducibility of semiquantification of striatal DAT availability is important for disease diagnosis, longitudinal monitoring of disease progression, and assessing the effectiveness of medication. Our study demonstrated that CT-guided ROI delineation could be a more reproducible method than manual SPECT ROI method. The manual SPECT ROI delineation is a simple method for semiquantifying striatal DAT availability and is widely used in clinical practice. It is easy to draw the contour of the striatum on SPECT in patients with normal tracer uptake. However, accurate ROI delineation may be a problem in patients with low tracer uptake or abnormal anatomy. Therefore, the interobserver variability tends to be high. Our results showed that manual SPECT ROI delineation could be an intraobserver reproducible method with moderate to substantial agreement for an experienced nuclear medicine physician, but the interobserver agreement was poor. As compared with the manual SPECT ROI method, the CT-guided ROI method had higher values of concordance correlation coefficient, closer biases to zero, and narrower limits of agreement in both intra- and interobserver analyses.

The intra- and interobserver variability introduced by manual interventions during the processing steps are intrinsic and cannot be eliminated. It can only be reduced by a highly trained observer and rigorous processing steps. Although CT-guided ROI delineation depends on manual processing, the manual processing steps can be more standardized to improve the reproducibility in a hybrid SPECT/CT system. First, ROIs were delineated based on morphological CT images rather than functional SPECT images, so the variability was stable regardless of the tracer uptake level. Second, the images reorientation is better standardized by determining the AC-PC line and midline of the brain on the fused SPECT/CT images. Therefore, the variability caused by differences in the realignment of the head was reduced. Finally, inspecting fused SPECT/CT images allows the physician to accurately delineate the striatal and reference ROIs, especially in patients with low specific bindings or anatomical changes from insults or a dilated ventricle.

Because there is no gold standard for semiquantitative striatal DAT availability, we used visual rating as standard for ROC curve analysis. Our results showed that both methods were highly accurate (AUCs ranged from 0.95 to 0.98). CT-guided ROI method had higher AUCs than manual SPECT method with borderline significance. We also used the clinical research acceptable method of software-based SPECT/MRI fusion for comparison. Although the striatal and subregional contours on CT are not as clear as those on MRI, our results demonstrated that semiquantifying striatal and subregional DAT availability using CT-guided ROIs correlated very well with the MRI-guided ROI method. In contrast, the correlation coefficients between manual SPECT and MRI-guided ROI methods were not as excellent as those between CT-guided and MRI-guided methods, especially in patients with low specific binding in the putamen. Since brain MRI is not always available to guide ROI delineation, CT-guided ROI method in a hybrid SPECT/CT system can be a feasible method in clinical routine practice. It is particularly useful for patients with low specific binding in the putamen, a common finding of Parkinson's disease. In addition, a hardware-based hybrid SPECT/CT system provides more accurate image coregistration than software-based image fusion of SPECT and MRI and the image processing is accessible in the manufacturer's nuclear medicine workstation without any extra software.

With the increasing availability of hybrid SPECT/CT devices, SPECT/CT is being used for many clinical indications. The advantages of SPECT/CT are better attenuation correction, increased specificity, and accurate depiction of the localization of disease and of possible involvement of adjacent tissue [25]. The main concern regarding the use of SPECT/CT in clinical practice is the additional radiation exposure from CT. An appropriate balance between the medical benefits of CT and risk produced by additional radiation exposure should be taken into consideration. The effective dose from CT in various nuclear medicine SPECT/CT studies has been reported from 0.06 to 11.9 mSv (2.3 to 666.4% increases in radiation dose compared with SPECT alone for Tc-99m tracer) [26]. In our study, the administered dose of Tc-99m TRODAT-1 is 925 MBq and the effective dose from the radiopharmaceutical is calculated as 11.1 mSv [27]. The mean CT effective dose was 0.36 mSv and the increase in radiation dose was very low at 3.2%. In addition to attenuation correction and added anatomical information for DAT-SPECT [28–30], our study demonstrated that CT can be used to guide ROI delineation for semiquantifying striatal DAT availability. It may also justify the extra radiation dose from CT.

Some limitations of this study have to be addressed. First, the benefit of using CT-guided ROI delineation in diagnostic performance and clinical impact was not evaluated. Second, the semiquantification is only based on Tc-99m TRODAT-1 SPECT/CT images. Data from other radioligands, such as I-123 agents, may need separate studies.

## 5. Conclusions

We conclude that CT-guided ROI delineation for semiquantifying striatal DAT availability in a hybrid SPECT/CT system is highly reproducible, and the semiquantitative data correlate well with data from MRI-guided ROIs. Therefore, CT-guided ROI delineation in a hybrid SPECT/CT system can be a feasible method for semiquantifying DAT availability in daily clinical practice.

## Figures and Tables

**Table 1:** Striatal, caudate, and putamen BP_ND_ from manual SPECT, CT-guided, and MRI-guided ROIs methods; the intra- and interobserver concordance correlation coefficients, Bland-Altman biases, and limits of agreement of manual SPECT and CT-guided ROI methods.

	Striatal BP_ND_	Caudate BP_ND_	Putamen BP_ND_
Manual SPECT ROI			
Intraobserver (observer A)			
First analysis	0.46 ± 0.24	0.55 ± 0.26	0.42 ± 0.24
Second analysis	0.48 ± 0.26	0.58 ± 0.29	0.38 ± 0.26
CCC	0.96	0.96	0.93
95% CI of CCC	0.93–0.98	0.94–0.98	0.89–0.96
Bias (mean difference)	−0.03	−0.03	0.03
Limits of agreement	−0.16, 0.10	−0.16, 0.11	−0.13, 0.20
Interobserver^*^			
Observer B analysis	0.39 ± 0.23	0.48 ± 0.22	0.36 ± 0.24
CCC	0.89	0.89	0.88
95% CI of CCC	0.82–0.93	0.83–0.93	0.80–0.93
Bias (mean difference)	0.07	0.07	0.05
Limits of agreement	−0.11, 0.25	−0.11, 0.25	−0.16, 0.27
CT-guided ROI			
Intraobserver (observer A)			
First analysis	0.49 ± 0.25	0.54 ± 0.25	0.46 ± 0.27
Second analysis	0.48 ± 0.25	0.52 ± 0.26	0.46 ± 0.27
CCC	0.98	0.97	0.97
95% CI of CCC	0.97–0.99	0.94–0.98	0.95–0.98
Bias (mean difference)	0.01	0.02	0.01
Limits of agreement	−0.08, 0.11	−0.11, 0.14	−0.12, 0.13
Interobserver^*^			
Observer B analysis	0.48 ± 0.26	0.56 ± 0.27	0.45 ± 0.28
CCC	0.97	0.95	0.96
95% CI of CCC	0.95–0.98	0.92–0.97	0.94–0.98
Bias (mean difference)	0.01	−0.02	0.02
Limits of agreement	−0.12, 0.13	−0.18, 0.13	−0.13, 0.16
MRI-guided ROI	0.46 ± 0.25	0.53 ± 0.26	0.43 ± 0.25

BP_ND_: nondisplaceable binding potential; SPECT: single photon emission computed tomography; ROI: region of interest; CCC: concordance correlation coefficient; CI: confidence interval; CT: computed tomography

^*^Interobserver analysis was compared with observer A's first analysis.
